# Improving sensitivity in the deep regions of a volume conductor using electrical focused impedance methods

**DOI:** 10.2478/joeb-2024-0012

**Published:** 2024-09-06

**Authors:** Mahjabin Mobarak, K Siddique-e Rabbani

**Affiliations:** 1 Southeast University, Dhaka, Bangladesh; 2 Department of Biomedical Physics and Technology, University of Dhaka, Dhaka, Bangladesh

**Keywords:** Electrical impedance, Tetrapolar Impedance Method (TPIM), Focused Impedance Method (FIM), Transfer Impedance, Deep probing of thorax

## Abstract

Bioimpedance measurements are becoming important in probing the human body for diagnosis and monitoring. An age old 4-electrode technique called tetrapolar impedance measurement (TPIM), giving transfer impedance, cannot localize a specific zone besides having large zones of negative sensitivity. A new technique named the focused impedance method (FIM) from Dhaka University (DU), Bangladesh used the algebraic average of two concentric and orthogonal TPIMs, localizing a zone of interest and having reduced magnitudes of negative sensitivity. Earlier, this was implemented with electrodes applied from one side of the human body giving information to shallow depths only. To get information from deeper regions, specifically, of the thorax, the same DU group placed two electrodes of a 4-electrode version of FIM at the front and two at the back in a horizontal plane of the thorax, using physics-based visualization. This was followed by a few quantitative studies using point sensitivity, which supported the concept. However, more quantitative studies still need to be performed, particularly using objects of finite sizes, in order to establish the technique on a stronger footing. The present study was taken up with this objective. A simplified approach was used in which the volume conductor was a rectangular non-conducting container filled with saline of uniform conductivity with an embedded spherical object – first an insulator and then a conductor. Electrodes were placed at specific chosen positions following the above visualization. Percentage change in transfer impedance with the object placed at different internal positions, compared to that without the object was obtained first using COMSOL simulation and then through experimental measurements. These were performed for both TPIM and FIM. The new configuration of 4-electrode FIM gave good depth sensitivity supporting the effectiveness of the new placement of electrodes.

## Introduction

Scientists around the world are working to develop simple, inexpensive, non-invasive techniques for human physiological research and diagnostics. Electrical impedance measurement techniques may have a significant role in this objective [1,2]. All body tissues have different electrical properties that change in health and disease [3]. Estimation of the electrical impedance of body tissues has been shown as a possible method for observing tissue and organ conditions. Besides, electrical impedance techniques are non-invasive, low cost, and straightforward, which offers a great advantage [4]. Again, there are organs whose electrical impedance changes with time (lungs, heart, stomach, etc.) and electrical impedance techniques could be used effectively to monitor these changes, identifying diseased or disordered conditions.

Two important electrical properties are evident in living tissues. Electrical conductivity due to free charges, and dielectric properties due to bound charges, the latter expressed by the parameter ‘permittivity’ [5]. Thus, electrical impedance is a representation of the electrical conductivity and permittivity and thus may distinguish heterogeneities within the body [6].

In a typical 4-electrode or tetrapolar impedance measurement (TPIM) technique, an alternating current of constant amplitude is injected into a volume conductor through a pair of electrodes and the resulting voltage across another electrode pair is measured. The ratio of the measured voltage to the applied current is said to give a parameter, ‘transfer impedance’ [7]. This is closely related to impedance defined through Ohm’s law, but an important difference is that Ohm’s law is defined using two electrodes only, which is not normally used in electrical bioimpedance measurement of tissues in the bulk region because of a high impedance at the tissue electrode (typically, skin-electrode) interface. TPIM essentially eliminates or minimizes the contribution of the tissue electrode interface to the measured impedance, providing a measure of the bulk impedance.

TPIM in a volume conductor does not give a good localization. Besides, it is associated with large negative sensitivity regions between the current and voltage electrodes, meaning that if the impedance of these regions increases, the total measured transfer impedance would decrease making interpretations difficult. That means if a TPIM measurement is carried out on a large organ, say the lungs, and if a significant part of it falls within the negative sensitivity region, then on inspiration when the impedance of the whole lung region increases, the measured change would be much less than expected, and depending on the electrode positioning, may even give a change in the opposite direction.

A group of researchers at the University of Dhaka proposed and implemented a new technique of electrical impedance measurement which they called the focused impedance method (FIM) [8-10]. For this, two TPIM measurements are taken orthogonally around a central zone of interest and the two are algebraically averaged to obtain the FIM. The total transfer impedance has increased sensitivity within the central zone, allowing measurement of changes in the transfer impedance of a specific target zone within the volume conductor, minimizing the effects of adjacent zones [11]. The negative sensitivity is also reduced compared to TPIM. FIM has three versions, having 8, 6 or 4 electrodes each with slightly different sensitivity patterns which may be used to advantage in studying different internal organs based on anatomical configuration.

**Figure 1:**
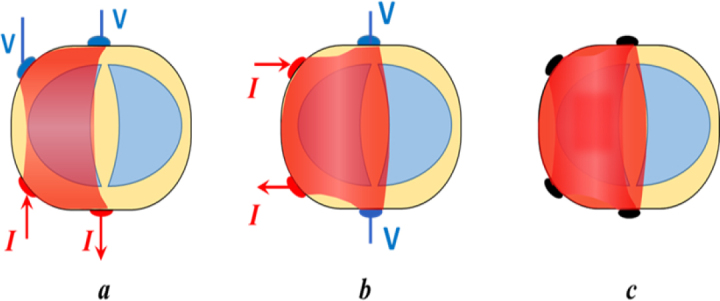
Schematic representation of the sensitive region on a transverse plane of a volume conductor for two orthogonal TPIMs (a, b) from which FIM-4 (c) is obtained. The current drive electrodes (red dots) and the potential electrodes (blue dots) are shown in the first two diagrams for the respective TPIM configurations [12].

For the application of FIM on the human body, most studies performed to date used electrodes on one side of the body giving rather shallow depth sensitivity. Recently, several configurations of electrodes for 4-electrode FIM (FIM-4) were proposed to increase the depth sensitivity of deep organs like lungs and stomach [12]. For this the author proposed placing two electrodes on the front of the body and two on the back, on a transverse (horizontal) plane as shown in Figure 1.

In a few subsequent publications, simulations were performed based on this idea for point sensitivity studies using a finite element software package named COMSOL Multiphysics®, for different electrode separations and geometries, demonstrating increased sensitivity in the deep regions [13,14]. These supported the predictions of the earlier publication (Reference 12). The same author later proposed a new six electrode TPIM which had two current electrodes at the two sides of the thorax and two pairs of potential electrodes at the front and the back with the corresponding ones being shorted. The advantage is that it can use simpler TPIM instrumentation [15]. This concept was also established through simulation and experimental studies on a phantom [16] and implemented for probing lungs and stomach [17,18].

Being able to monitor deep organs using electrical impedance provides many advantages, one of which is the ability for continuous monitoring. Besides, it is noninvasive, non-hazardous and low-cost. Probing deep organs like lungs is very important in measuring any disorders like edema, pneumonia, lung cancer, etc.

Earlier studies to evaluate the efficacy of the 4-electrode FIM configuration as suggested in [12] to monitor deep regions were mostly carried out using point sensitivity, which supported the concept. However, more quantitative studies still need to be performed, particularly using objects of finite sizes, to establish the technique on a stronger footing. The present work was taken up with this objective. It studied the overall sensitivity to transfer impedance due to a small spherical object by placing it at different positions inside a volume conductor with uniform conductivity. The study was performed for an insulating as well as a conducting object, both using finite element simulation (using the COMSOL Multiphysics® software package) and experimental measurements on a phantom. The main idea of this study was to see whether the changes in impedance using a finite sized object have a qualitative agreement to the basic concept of the measurement, i.e., enhancing the sensitivity in the deep regions of a volume conductor due to a particular electrode placement [12] and to the related point sensitivity studies carried out earlier [13,14].

## Materials and methods

### Basic description

For the numerical simulation and experimental phantom studies to be described below, the geometries of the volume conductor, the electrodes and the inserted objects were kept the same as far as possible.

The volume conductor (phantom) chosen was of 33 cm length, 26 cm width, and 12 cm height. These dimensions were kept the same as used for the earlier studies [13, 14] so that the results are comparable. The phantom was filled up with saline of conductivity (σ) 0.081 S/m, which was the background conductivity of the volume conductor. A measurement frequency of 5 kHz was chosen for both simulation and experimental studies. Relative permittivity of water was taken as 80. For the experimental study a spherical rubber ball of 4.2 cm diameter was used as an insulating object. To get a conducting object of the same size, aluminum foil was wrapped around the same rubber ball. Therefore, for the corresponding numerical simulation, objects with the same diameter, 4.2 cm, were assumed. The conductivities were taken as 1×10^-5^ S/m for the insulating sphere (here, rubber), while that of the conducting sphere (here, aluminum) was taken as 3.8 × 10^7^ S/m. The corresponding relative permittivities were taken as 7 and 3.5, respectively for the frequency chosen (5 kHz). Though the relative permittivity changes with frequency, we have implemented only one frequency in this case. Moreover, for this type of simplified model the relative permittivity does not have any significant effect on the measured results, so we chose values which are typically quoted for these materials.

**Figure 2:**
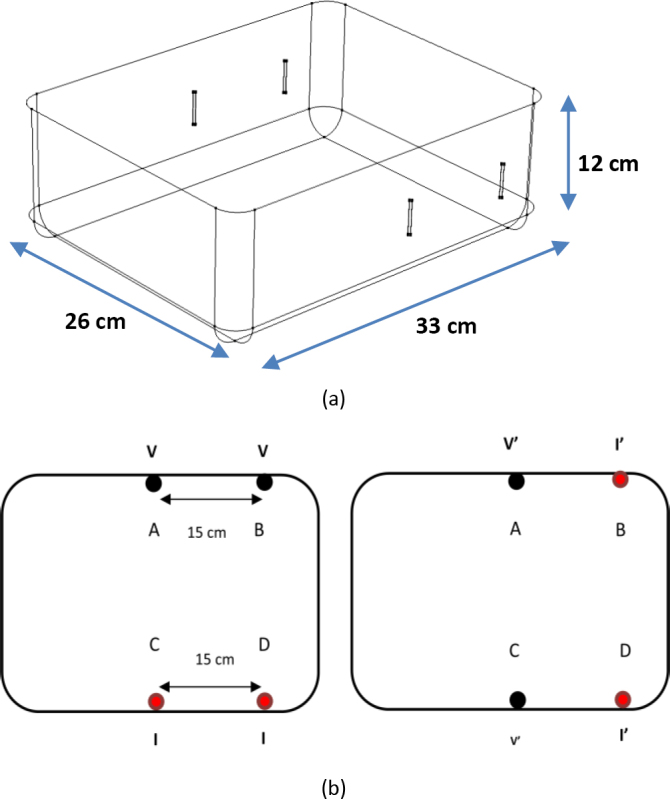
(a) A 3D perspective of a rectangular phantom (33 cm × 26 cm × 12 cm) showing the placement of electrodes on the two opposite sides. (b) Cross sectional view showing schemes for 4-electrode configuration showing current and voltage electrodes: 1^st^ step – measuring TPIM1 (left hand figure), 2^nd^ step – measuring TPIM2 (right hand figure), changing the electrode connections indicated by I’ (current drive, red) and V’ (potential electrodes, black), respectively. FIM was obtained as an algebraic average of TPIM1 and TPIM2.

For the experimental studies, 3 cm long stainless-steel rod electrodes with a circular cross section of 0.15 cm radius were placed vertically, centered inside the side walls of the phantom as shown in Figure 2(a). For the numerical simulation, electrodes with the same shape and cross section were used. For the simulation, the conductivity of the electrodes was taken as 4.6x10^6^ S/m and relative permittivity was taken as 1.

Separations between the two electrodes at the front as well as that for the two electrodes at the back were taken as 15 cm, as shown in Figure 2(b). This means that the area bounded by these electrodes has a dimension of 15 cm x 26 cm. For an ideal 4-electrode FIM, the area should have been a square, however, we have made this deviation deliberately to accommodate the ultimate objective of the present study, i.e., to study deep regions of individual lungs. The current drive and voltage electrodes for implementation of the two orthogonal TPIM measurements (TPIM1 and TPIM2) are also shown, the red-colored ones for the current drive and the black ones for voltage. The algebraic average of the above two values was taken as the corresponding 4-electrode FIM value.

For the present study, each object was placed at different positions (same for both simulation and experimental) inside the volume conductor.

**Figure 3:**
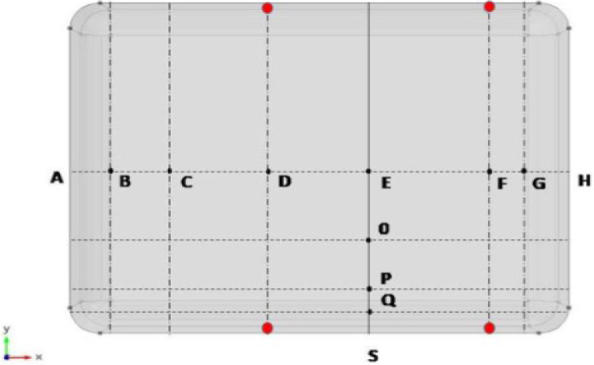
A cross-sectional view of the modelled phantom showing the points where the centers of the spherical objects were placed (black dots, with lettered names). Along the X-axis, AB = 3 cm, AC = 6.5 cm, AD = 13 cm, AE = 20.5 cm, AF = 28 cm, AG = 30 cm, and along the Y-axis, SQ = 2.1 cm, SP = 3.25 cm, SO = 6.5 cm and SE = 13 cm. Red dots show the positions of the electrodes.

Figure 3 shows the horizontal cross-sectional view of the modelled volume conductor (phantom). Points B, C, D, E, F, and G along the X-axis and points E, O, P, and Q along the Y-axis show the positions where the centers of the spherical objects were placed for this simulation study. The geometrical positions of these points are specified in the figure caption.

**Figure 4:**
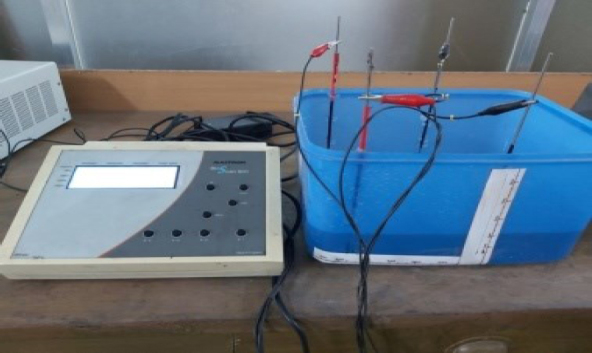
Phantom with electrodes and the instrument for electrical impedance measurement (Maltron Bioscan 920-II) used for the experimental study.

An experimental phantom study similar to that of the simulation study mentioned above was also performed. For this, a plastic box filled with saline was used as the experimental phantom. It was filled with saline to a height so that the saline volume had the same dimensions as that used for the simulation. Electrodes were made using long steel rods, insulating their upper ends and keeping a length of 3 cm on the lower side bare. These were then fixed inside the phantom at the desired positions. As mentioned before, for an insulating object, a rubber ball of 4.2 cm diameter was used. For a conducting object, the rubber ball was wrapped with aluminum foil. A commercial electrical impedance measuring device (Maltron Bioscan 920-II) was used for the measurements. Figure 4 shows the measurement set up in the laboratory.

### Analysis for simulation

Point sensitivity *S* in TPIM [19] can be found using the following equations, assuming unit current densities,


1 $$S=\frac{\vec{J_{1}}\cdot \vec{J_{2}}}{I^{2}}$$

2 $$S=\frac{J_{1}\cdot J_{2}\;\text{cos}\theta }{I^{2}}$$

where **J**_1_ and **J**_2_ represent the current density vectors at the point in question due to current driven through the two pairs of electrodes, the current drive pair and the potential electrode pair, I is the magnitude of the current (equal through both pairs) and θ is the angle between the two vectors. In our work the current was taken as 1 A.

For the simulation study, firstly, the impedance of the phantom (*Z_p_*) without any inserted object was obtained using the following equation,


3 $$Z_{p}=\int \rho_{b}S\;dV_{p}$$

where *ρ_b_ is* the resistivity of the background liquid, *S* is the appropriate point sensitivity, the integration being carried out over the phantom volume *V_p_*.

When an object of a different resistivity (*ρ_ob_*) is inserted at any specific point of the volume conductor, the impedance contribution of the object (*Z_ob_*) was obtained using the following equation, integrated over the object volume, *V_ob_*.


4 $$Z_{Ob}=\int \rho_{ob}S\;dV_{ob}$$

Under this situation, the impedance of the phantom region (*Z_n_*) outside the inserted object was obtained using the following equation,


5 $$Z_{n}=\int \rho_{b}S\;dV_{n}$$

Where the background resistivity *ρ_b_* is used and the integration is performed over the volume *V_n_* outside the object.

The total transfer impedance *Z_T_*, as measured with an inserted object, would then be given by the sum of the above two,


6 $$Z_{T}=Z_{n}+Z_{ob}$$

To determine the change of impedance (*ΔZ*) for inserting the object at various positions the following equation is used, which is expressed in a percentage form.


7 $$\Delta Z\%=\frac{\left(Z_{T}-Z_{p}\right)}{Z_{p}}\times 100\%$$

### Ethical approval

The conducted research is not related to either human or animal use.

## Results and observations

**Figure 5:**
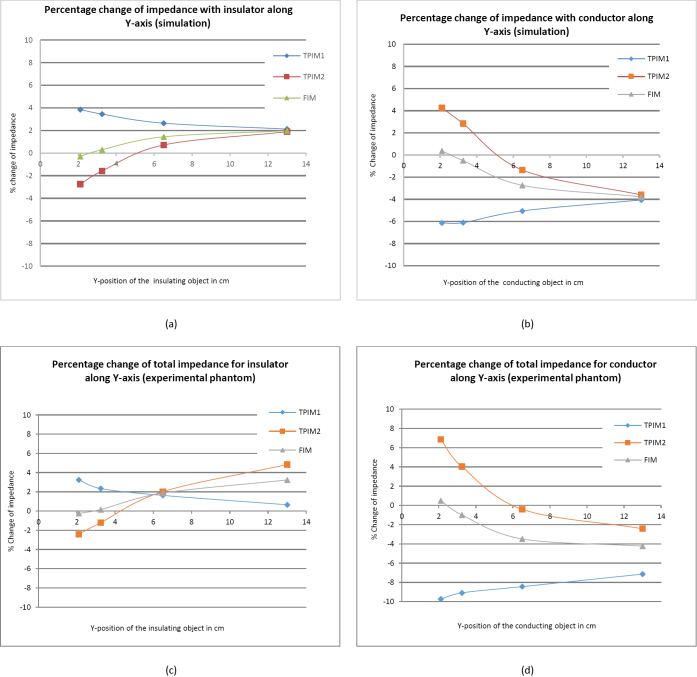
Simulation results of the percentage change of impedance for TPIM1, TPIM2, and FIM with a spherical insulator (a) and conductor (b) placed at different points along the width of the Phantom (Y-position). The same for the experimental phantom(c) with insulator and (d) conductor.

Figure 5 shows the percentage changes of the total transfer impedance values (TPIM1, TPIM2 and FIM) due to the positioning of the two objects at different positions along the Y-axis as represented by E, O, P, and Q in Figure 3 for both simulations [Figures 5(a) and 5(b)] and experimental studies [Figures 5(c) and 5(d)]. These show the transfer impedances (will also be referred to as ‘impedance’ interchangeably in this paper) obtained for different Y-positions, E, O, P, and Q (Figure 3) of the target object for both insulating and conducting objects following electrode configurations and placement described in Figure 2 and 3.

The position along the X-axis was kept fixed at 20.5 cm, the center point between the electrodes, while Z was fixed at 6 cm, the mid position along the height. As a symmetrical configuration is taken along the Y-direction, positions from the center of the box to one side only were analyzed. FIM was obtained as an algebraic average of the corresponding TPIM1 and TPIM2 respectively.

These outputs pertain to conductivity values of 1 × 10^-5^ S/m for the object and 0.081 S/m for the background as mentioned before.

However, it is slightly higher near the electrodes (surface of the volume conductor) for TPIM1 while for TPIM2, it has a negative value, completely opposite to what is expected from an insulated object. This is because of the negative sensitivity mentioned earlier. In Figure 5(b) for a conducting object the nature of the results is similar, TPIM2 giving values opposite to that expected for a conducting object when placed near the electrodes, due to negative sensitivity.

In both cases (insulating and conducting objects) FIM has positive sensitivity almost over the whole measured range except for a negligible negative sensitivity near the electrodes. The sensitivity is highest at the center.

From Figure 5(a) for an insulating object, it may be observed that the percentage change of impedance at the center is almost the same for TPIM1, TPIM2 and FIM.

Figure 5(c) should correspond to the simulation results shown in Figure 5(a) and the trends for each graph individually correspond well. However, while in Figure 5(a) the three graphs coincided in the middle, corresponding to Y = 13 cm, here the coincidence occurred earlier, at about 6 cm. Again, TPIM2 shows negative sensitivity near the electrodes while that of FIM is very small. Figure 5(d) for a conducting object also shows similar trends as in Figure 5(b). Again, in Figure 5(d) the graphs do not coincide within the range of the measurement while these coincided in Figure 5(b). Like Figure 5(b), Figure 5(d) shows negative sensitivity for TPIM2 (positive change for a conducting object) near the electrodes while it is very small for FIM.

The differences may occur due to differences in the experimental phantom parameters compared to those used for numerical simulations and in the placement of the objects in the specified positions.

**Figure 6:**
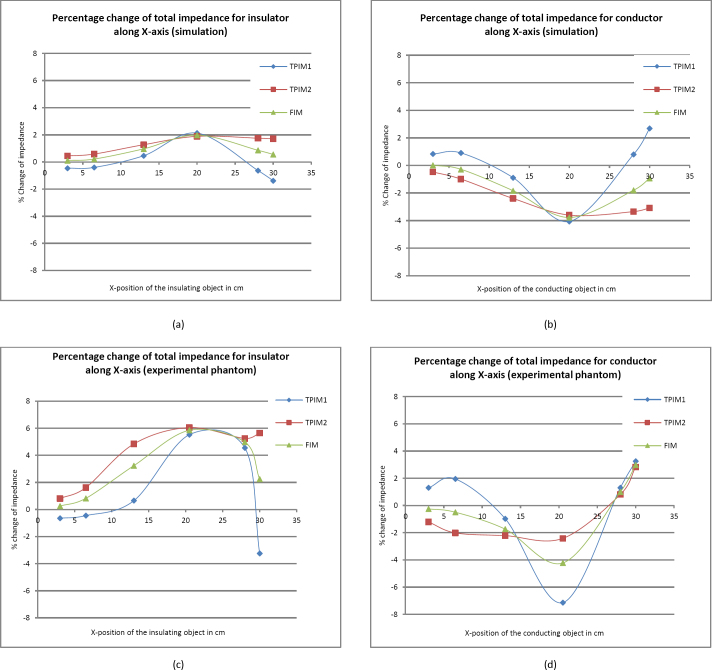
Simulation results of the percentage change of impedance for TPIM1, TPIM2, and FIM with a spherical insulator (a) and conductor (b) placed at different points along the width of the Phantom (X-position). The same for the experimental phantom(c) with insulator and (d) conductor.

Next, the spherical object’s placement was varied along the length of the phantom (along the X-axis) keeping the Y value fixed at 13 cm, the mid-point of the phantom (points B, C, D, E, F, G in Figure 3). The Z-coordinate of the center of the object was fixed at Z = 6 cm, as before. The percentage changes of total impedance for the insulating and conducting objects are shown in Figure 6. Here the electrodes were placed at 13 cm and 28 cm along the X-axis, 20.5 cm being the center of the configuration.

From figures 6(a) and 6(b) it may be observed that as the object was moved from left to right the change in transfer impedance for all the three measurements (TPIM1, TPIM2, and FIM) went through a peak at around 20.5 cm, the center point of the electrode. However, here TPIM1 shows negative sensitivity near the right and left (lower and higher values of X) while there is no negative sensitivity with TPIM2. However, there is no negative sensitivity with FIM, and it shows a greater variation around the peak, showing improved localization of the object.

The experimental results shown in Figures 6(c) and 6(d) correspond to the simulation results shown in Figures 6(a) and 6(b), respectively. Here the trends for each graph individually match well. However, percentage change of the experimental graphs is slightly higher than that of the simulation results near the central position of the electrodes.

## Discussion

The study essentially attempts a quantitative evaluation of the depth sensitivity that can be achieved using the electrode configuration for FIM suggested in reference 12 and to show whether the results expected in this publication match quantitative outcomes.

As mentioned before, this present study comprised both a finite element simulation using the COMSOL Multiphysics® software package and an experimental study using a simple phantom. In order to have a good correspondence between the two methods all the parameters were kept as close as possible. This includes the dimensions and the conductivities of the volume conductor and the embedded objects, the electrodes and their placement.

The embedded spherical objects, a conductor and an insulator, were chosen to be of the same dimension in both studies. However, since the conducting object was made by wrapping aluminum foil on a rubber ball, the dimensions differed slightly from those in the numerical analysis. Again, the conductivities chosen for the two types of objects (aluminum as a conductor and rubber as an insulator) for the simulation may not match exactly those used in the experiment. However, this should not cause a big difference. The conductivity of the volume conductor in the experimental phantom was taken as 0.081 S/m, the value obtained by measuring the conductivity of tap water with the addition of a little salt. The same conductivity was also used for the simulation study. However, with time, the conductivity of the experimental phantom may change slightly due to evaporation. Cylindrical rod electrodes (rods with a circular cross section) of 3 cm in length with a diameter of 0.3 cm were implemented for both simulation and phantom studies. The current density may vary due to the size and shape of the electrodes and this is a source of error between the simulation and the experimental measurement. Also, it may not always be possible to achieve an accurate placement of the object. All of the above may cause some discrepancies between the experimental results and those of the simulated study.

The simulated measurements were performed placing each object (one at a time) at different specified positions within the phantom which provided an idea about how it affected the total transfer impedance measurement in both the electrode configurations studied. As the volume of the object was very small compared to the volume of the phantom, the variation of the impedance was small, which was expected, but this study provided a visualization of the expected change in bioimpedance for objects of finite dimensions. Negative point sensitivity is natural to expect in the region between current and potential electrodes for TPIM. A finite sized object as used here will integrate the sensitivity over its volume, which may contain points with both negative and positive sensitivity. So, the effects may be different from that for point sensitivities alone.

From Figure 5(a) for object placement from the frontal surface towards deep regions (Y direction) it was observed that for TPIM1 (reference: Figure 2(b)) the change in impedance due to the insulating object is highest near the surface and decreased from about 4% at 2 cm to about 2% at the center (13 cm), while for TPIM2 the change in impedance near the surface was negative, about -3% at 2 cm, which became positive at a distance of about 5 cm from the surface, gradually increasing to about +2% at the center. This shows the weakness of simple TPIM because of its negative sensitivity, which occurs in the regions between the current (I) and potential or voltage (V) electrodes. On the other hand, the negative value of FIM is very small near the electrodes, being much less than that obtained with TPIM2. This shows the strength of FIM. Figure 5 (b) for a conducting object showed opposite results which is expected, and the essential features are nearly the same.

Experimental results shown in Figure 5(c) and (d) also match the trends of the simulation results though the percentage change is slightly on the higher side. Clearly, Figure 5 shows that the percentage change in FIM is higher at the center, indicating the success of this electrode technique, configured to increase the sensitivity near the center of a volume conductor, i.e., in the deep region. Also, a low percentage change in impedance was observed near the front surface because of the cancellation of negative and positive sensitivity values. However, FIM became positive at about 2.5 cm from the surface and increased to about 2% at the center. Moreover, from about 8 cm from the frontal surface to the center (13 cm from the frontal surface), i.e., for a distance of about 5 cm the change in impedance was small. From symmetry considerations, this indicates that for twice this distance, i.e., for about 10 cm around the center from the front to the back of the 26 cm deep phantom, the change in impedance was small. This means, FIM gives an almost uniform sensitivity over a large volume near the center, which is a big advantage for studies of objects at depth, such as the lungs. This shows that 4-electrode FIM as configured was successful in giving information from the deeper regions.

However, TPIM1 may also be used if one can ensure that the position of the object is along the Y direction, through the center point of the electrode configuration. However, TPIM2 cannot be used for such positions because of high negative sensitivities near the front and the back. Therefore, for arbitrary positioning of objects in the deep regions, TPIM cannot give good results, but FIM can. Again, TPIM1 gives high negative sensitivity values along the X-axis at both the left and right ends of the phantom (Figure 6) while TPIM2 does not have negative sensitivity values. FIM does not demonstrate negative sensitivity in the simulation but shows some negative sensitivity at the right end of the experimental phantom for the conducting sphere (Fig. 6d), but not for the insulating one (Fig. 6c). However, all of these show a high sensitivity at x = 20.5 cm, the center of the electrode configuration. Again, because of the above-mentioned arguments, FIM is better for the arbitrary positioning of objects.

The peak value obtained at 20.5 cm shows that this arrangement may separate values from the left and right lungs, which is very important in terms of clinical measurements. By judicial placement of electrodes, one may get more sensitivity from either the left or the right lung.

For the conducting object, the impedance values were essentially the opposite, but the patterns were similar. It needs to be mentioned that the conductivity ratios of the two objects to that of the background (of saline) are different, varying by two orders of magnitude, due to which the differences were observed.

The results of the experimental measurements produced similar patterns as those obtained using simulation although specific values were somewhat different. This was possibly due to differences in the dimensions and placement of objects compared to that in the simulation.

This study supports the methods proposed by Reference 12 for the measurement of deep regions of the human body, particularly for the lungs using a modified configuration of the 4-electrode FIM. Further studies should be taken up using improved thorax models and on real life subjects with and without lung problems.

The model which we have studied is very simple. Besides, the size of the inserted object was small (4.2 cm) compared to organs like the lungs, which was the main focus of this study. This small object gave a change of a few percent. Therefore, we would expect much larger changes in practical measurements with lungs as the target organs.

The large size of the lungs also requires that large parts of it do not fall in the negative sensitivity zones. If this happens, the real variation of impedance with breathing will be reduced, giving an erroneous assessment and diagnosis. This shows the strength and benefit of the FIM over TPIM since FIM has less negative sensitivity.

A human lung is surrounded by many organs and tissues with complex impedance distributions and a question may be raised on the usefulness of studies such as this one on very simple volume conductors. Actually, most of the measurements of lungs that we will be looking for are lung ventilation and lung perfusion. These are dynamic processes where the impedance of the lung only changes, not of the organs and tissues lying outside. Therefore, when one is measuring changes in impedance between inspiration and expiration, it is the changes in the lungs only which will be registered in the measurement. Again, for certain conditions such as cancer, the impedance and its variation with frequency are expected to vary between healthy and diseased tissues. There again, such variations may be registered using FIM measurements.

Again, the placements of the electrodes are very important when we target a particular organ from the outside. Understanding the basic physics will help in the choice of electrode placements for useful FIM measurements.

The present study has enhanced the understanding of the 4-electrode FIM with electrodes at the front and back of the thorax in obtaining information from the deep regions of the lungs. We hope FIM will soon become a popular modality for studies of deep organs like lungs, stomach, heart etc., enhancing healthcare globally.
